# Pneumonia in Parkinson's disease: barium aspiration in videofluoroscopic swallowing study

**DOI:** 10.1002/rcr2.546

**Published:** 2020-03-01

**Authors:** Kwang‐Hwa Chang, Yu‐Tien Tzeng, Jing‐Hwa Wey, Yuan‐Jun Liu, Yen‐Nung Lin, Wen‐Kuei Chung

**Affiliations:** ^1^ Department of Physical Medicine and Rehabilitation, Wan Fang Hospital Taipei Medical University Taipei Taiwan; ^2^ Graduate Institute of Injury Prevention and Control, College of Public Health and Nutrition Taipei Medical University Taipei Taiwan; ^3^ Taipei Neurological Institute Taipei Medical University New Taipei City Taiwan; ^4^ Division of Pulmonary Medicine, Department of Internal Medicine, Wan Fang Hospital Taipei Medical University Taipei Taiwan; ^5^ Department of Neurosurgery, Wan Fang Hospital Taipei Medical University Taipei Taiwan

**Keywords:** Aspiration, dysphagia, Parkinson's disease, pneumonia, videofluoroscopic swallowing study

## Abstract

Barium aspiration into the tracheobronchial tree is a remarkable warning sign of dysphagia‐related aspiration pneumonia. Clinical swallowing assessment is warranted for patients with aspiration pneumonia and videofluoroscopic swallowing study is a good add‐on tool for dysphagia treatment plan. In patients with Parkinson's disease, dysphagia is often overlooked due to atypical presentations.

## Graphical abstract

This is an excellent teaching image for daily clinical practice. Barium aspiration into tracheobronchial tree is a remarkable warning sign of dysphagia‐related aspiration pneumonia. In patients with aspiration pneumonia, a thorough swallowing function assessment shall be considered. Also, along with clinical assessment, videofluoroscopic swallowing study is a good add‐on tool for dysphagia treatment plan and shared decision‐making for nasogastric tube placement to patients and their caregivers. Owing to atypical presentation in patients with Parkinson's disease, dysphagia is often overlooked. Therefore, extra attention should be paid.

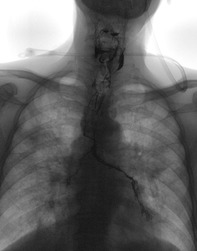

## Clinical Image

**Figure 1:**
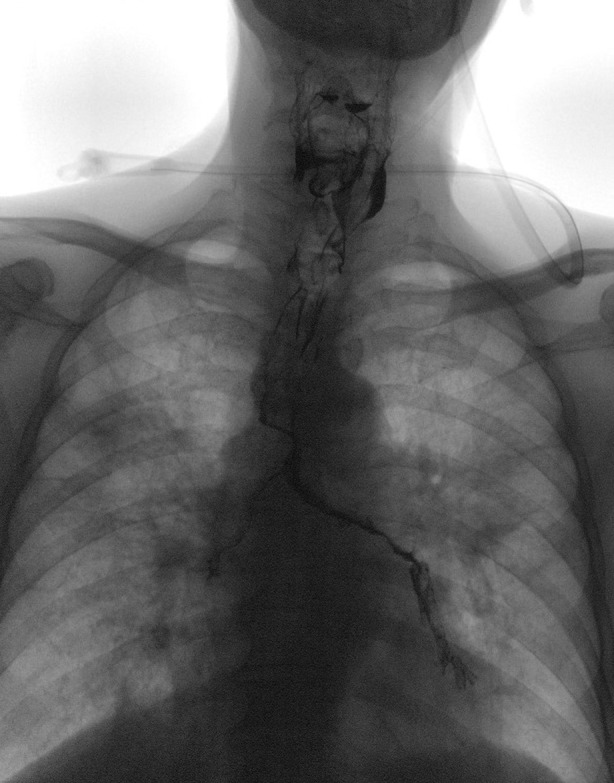
Barium retention in the tracheobronchial tree, more on the left side and the pyriform sinus in the hypopharynx on videofluoroscopic swallowing study (VFSS).

A 78‐year‐old male patient with Parkinson's disease was hospitalized because of pneumonia. He choked on liquids and solid foods progressively in the recent six months. Clinical swallowing assessment revealed pre‐ and post‐swallowing wet voice and delayed swallowing reflex. The patient also received videofluoroscopic swallowing study (VFSS) to assess the dynamic swallowing process in oral, pharyngeal, and oesophageal phases 1. The chest radiography immediately after VFSS revealed aspiration of barium into his bilateral bronchial trees with a more prominent aspiration on the left side and at bilateral pyriform sinus in hypopharynx (Fig. 1). Rather than right‐sided bronchial tree barium dislodgement predominance in most of the cases, left‐sided predominance can be related to poor trunk control and truncal deviation in Parkinson's disease. For dysphagia, our speech–language pathologist provided swallowing training to strengthen oral and pharyngeal muscles, stimulate swallowing reflex, and promote safe swallowing technique. After a two‐week treatment of aspiration pneumonia, the patient was discharged with his nasogastric tube remained. We also educated the patient's caregiver and family for the necessity of nasogastric tube placement at the moment and swallowing training for severe dysphagia in this patient, and also informed them about the risk of aspiration in oral feeding.

### Disclosure Statement

Appropriate written informed consent was obtained for publication of this case report and accompanying images.
